# Towards the eradication of HPV infection through universal specific vaccination

**DOI:** 10.1186/1471-2458-13-642

**Published:** 2013-07-11

**Authors:** Piergiorgio Crosignani, Antonella De Stefani, Gaetano Maria Fara, Andrea M Isidori, Andrea Lenzi, Carlo Antonio Liverani, Alberto Lombardi, Francesco Saverio Mennini, Giorgio Palu’, Sergio Pecorelli, Andrea P Peracino, Carlo Signorelli, Gian Vincenzo Zuccotti

**Affiliations:** 1 Obstetrics and Gynaecology Clinic, Università degli Studi di Milano, Milan, Italy; 2 ENT Department, Ospedale Mauriziano, Turin, Italy; 3 Department of Public Health and Infectious Diseases, Sapienza Università di Roma, Rome, Italy; 4 Department of Experimental Medicine, Sapienza Università di Roma, Rome, Italy; 5 Department of Experimental Medicine, Section of Medical Pathophysiology, Food Science and Endocrinology, Sapienza Università di Roma, Rome, Italy; 6 Preventive Gynecologic Oncology Unit - Department of Mother and Infant Sciences, Università di Milano, Foundation IRCCS Ca’ Granda Ospedale Maggiore Policlinico, Milan, Italy; 7 Fondazione Giovanni Lorenzini Medical Science Foundation, Milan, Italy; 8 CEIS Sanità - Centre for Health Economics and Management (CHEM) Faculty of Economics and Faculty of Science, University of Rome “Tor Vergata”, Rome, Italy; 9 Faculty of Statistics, University of Rome La Sapienza, Rome, Italy; 10 Institute of Leadership and Management in Healths, Kingston University, London, UK; 11 Department of Molecular Medicine, Università di Padova, Padua, Italy; 12 Department of Mother and Infant Sciences and Biomedical Technologies - Rector, Università di Brescia, Brescia, Italy; 13 Giovanni Lorenzini Medical Science Foundation, Houston, TX, USA; 14 Department SBIBIT, Università di Parma, Parma, Italy; 15 Department of Pediatrics, Università degli Studi di Milano - Luigi Sacco Hospital, Milan, Italy

**Keywords:** HPV infection, Condylomas, Cervical cancer, Genital cancer, Oro-pharyngeal cancer, Anti-HPV vaccines, Universal vaccination, Vaccination programs, Incremental cost-effectiveness ratio

## Abstract

**Background:**

The Human Papillomavirus (HPV) is generally recognized to be the direct cause of cervical cancer. The development of effective anti-HPV vaccines, included in the portfolio of recommended vaccinations for any given community, led to the consolidation in many countries of immunization programs to prevent HPV-related cervical cancers. In recent years, increasing evidence in epidemiology and molecular biology have supported the oncogenic role of HPV in the development of other neoplasm including condylomas and penile, anal, vulvar, vaginal, and oro-pharyngeal cancers. Men play a key role in the paradigm of HPV infection: both as patients and as part of the mechanisms of transmission. Data show they are affected almost as often as women. Moreover, no screening procedures for HPV-related disease prevention are applied in men, who fail to undergo routine medical testing by any medical specialist at all. They also do not benefit from government prevention strategies.

**Discussion:**

A panel of experts convened to focus on scientific, medical, and economic studies, and on the achievements from health organizations’ intervention programs on the matter. One of the goals was to discuss on the critical issues emerging from the ongoing global implementation of HPV vaccination. A second goal was to identify contributions which could overcome the barriers that impede or delay effective vaccination programs whose purpose is to eradicate the HPV infection both in women and men.

**Summary:**

The reviewed studies on the natural history of HPV infection and related diseases in women and men, the increasing experience of HPV vaccination in women, the analysis of clinical effectiveness vs economic efficacy of HPV vaccination, are even more supportive of the economic sustainability of vaccination programs both in women and men. Those achievements address increasing and needed attention to the issue of social equity in healthcare for both genders.

## Background

Human Papillomavirus (HPV) infection is the most widely spread sexually transmitted infection in some areas of the world, with up to 70% of the population expected to become infected at some point of their lifetime [1,2]. The majority of these infections are subclinical, unrecognized, and benign [3,4]. Since HPV was discovered to be the direct cause of cervical cancer [5,6], scientific data paired to the development of effective anti-HPV vaccines, accepted by health organizations, and included in the portfolio of recommended vaccinations for the community, have led to the consolidation in many countries of immunization programs to prevent HPV-related cervical cancers. Knowledge and experience accrued thus far, support and better address prevention programs in utilizing HPV vaccination for the benefit of the community. HPV infection, earlier correlated only to cervical carcinoma, today is acknowledged to be primarily responsible for cancerous and precancerous lesions of the genital area in both males and females and, in a lower percentage but with a not indifferent burden, of head and neck cancers [7,8]. Although HPV infections are known to be mainly a sexually transmitted disease, recent studies in non-sexually abused children infected with HPV suggest different forms of transmission [9,10]. Reports on the non-sexual transmission of anogenital warts, e.g. by prenatal mode, show the importance of maternal gynecologic history [11], and can help to understand better suspected sexual abuse in children [12]. Hand-genital transmission in adults should also be considered as a non-sexual means of transmission of HPV [13,14] although it has yet to be confirmed [15].

Until today, HPV prevention strategies through vaccination have targeted women mainly against cervical cancer. HPV vaccination, as efficacious means to reduce the development of cervical cancer in women in primary intervention strategies, has already been shown to be highly effective in reducing HPV-related lesions, such as genital warts as well as CIN 2/3 [16,17]. The significant declines in the proportion of young women found to have genital warts and the absence of genital warts in vaccinated women in 2011 suggests that the human papillomavirus vaccine has a high efficacy outside of the trial setting. Vaccination is undoubtedly a primary prevention tool; furthermore the expected eradication of the most prevalent HPV types will decrease the need of intense screening (secondary prevention) and cervical excisions for high grade disease (tertiary prevention). Now increasing evidence demonstrates how important the burden of HPV-correlated diseases also is in men. Epidemiological data show that in Europe and the USA, the burden of HPV-related head and neck cancers is carried mainly by men (4 times more than women), which shows that males are more than mere vectors [18,19]. Between 2006 and 2007 many countries have implemented HPV vaccination programs only for girls around 12 years of age. In the beginning the two available and approved vaccines were intended to target females only. After the approval of the quadrivalent vaccine (HPV4) indications for men, the USA, Canada, and Australia now recommend routine vaccination for both men and women. Men however are not yet included in nationally funded routine HPV vaccination programs in Europe (except Austria) and in many other countries.

Data on disease burden, vaccine efficacy, vaccine safety, cost-effectiveness, and social and ethical factors need to be taken into consideration when authorities decide to add men to European HPV vaccination programs. This paper summarizes the topics debated by a panel of experts convened to focus on scientific, medical, and economic studies, and on achievements from health organizations’ intervention programs on the matter. The goal is to better develop a knowledge platform to be used to further support and promote eradication of HPV infections in both women and men. The discussion was structured to identify contributions which could overcome common barriers that impede or delay effective vaccination programs whose purpose is to eradicate the HPV infection in both women and men.

## Discussion

### Natural history of HPV infection in women and men

The increased understanding of the natural history of HPV infection recently supported one of the main breakthroughs of medical science. HPVs are double-stranded DNA viruses that replicate within stratified squamous epithelia that need micro-abrasions or areas of transitional epithelium, such as in the cervix, anus, and tonsils, to be able to infect epithelial cells [20]. After infection, the virus makes use of the cells’ normal DNA replication machinery to produce further viral genetic fragments at the supra-basal layer of the epithelium [20,21]. Like all papillomaviruses, HPVs establish productive infections only in keratinocytes of the skin or mucous membranes. While the majority of the known HPV infections cause no symptoms in most people and are usually spontaneously cleared by the host, some types can cause warts while others may lead – in a few cases – to cancers of the cervix, vulva, vagina, penis, anus, and oro-pharynx. More than 130 genotypes of HPV have been described; types are divided into high risk and low risk according to their ability to produce benign or malignant lesions over time. The main high risk HPV types, classified as carcinogenic to humans by the International Agency for Research and Cancer (IARC) since 1995 [22], are HPV types 16 and 18, which are responsible of about 70% [2,23] of cervical cancers worldwide and most HPV-related pre-cancerous lesions in other anatomical regions whether genital or not [24,25]. Among the low risk types, HPV types 6 and 11 account for approximately 90% of genital warts. Seventy percent of infections are usually cleared after one year and 90% in two years [20,26]. However, when the infection caused by high risk (HR) genotypes of HPV, persists – in 5% to 10% of infected women – there is a high probability of developing precancerous lesions of the cervix, which can progress to invasive cervical cancer. This process usually takes 10-15 years, providing many opportunities for prevention, detection, and treatment of the pre-cancerous lesion involved [21]. Several models show that what one may consider today as remission of infection might not be remission at all [20]. As already mentioned, increasing evidence in epidemiology and molecular biology have supported the oncogenic role of HPV in the development of other genital cancers including penile, anal, vulvar, and vaginal cancers [27,28] and some oro-pharyngeal cancers [8,29-31] that share mucosal junction similarities, such as the anal and cervical areas [32]. Several risk factors seem to affect HPV infection, from the number of sexual partners [33,34] and oral contraceptive use [35] to smoking and alcohol: the latter ones particularly related to head and neck cancers [36]. Other risk factors have been studied including condom use [37] and circumcision [38,39]. In women, evidence suggests two HPV incidence high peak points at < 25 and around 45 years of age [40]. On the other hand, in men, HPV prevalence and incidence seem to be constantly high at all ages [41,42]. The most common lesions in both sexes are anogenital warts (AGW), mainly attributable to HPV types 6 and 11 (> 90%). Of these cases, 20-50% involve co-infection with other high risk HPV types [27,43]. In fact, studies have demonstrated that both in females and males, AGW patients have a higher risk of developing HPV-related cancers [44]. These results seem to contradict statements from the American Centers for Disease Control (CDC) (2010) which indicate that AGW, except in very rare and unusual cases, will not turn in cancer. The types of HPV that cause AGW are different from the types that can cause anogenital cancer; however, subjects with low risk (LR) HPV types should be considered at higher risk of having cancer by HPV 16 in the future. Knowledge of natural history and epidemiology of HPV in men is constantly increasing although it still remains less extensive than in women. Cancers related to HPV diagnosed every year in males have been shown to be approximately half the number of HPV-related cancer cases in women. This proportion of 1:2 (not 1:100 or 1:1000) is significant, without taking into account other less severe HPV-related diseases that have a higher incidence and life-long-prevalence in men than in women. HPV-16 and -18 are found to account for 90% of all HPV-related cancers in men [45]. The most recent studies in Europe show that 17,403 cancer cases in men are expected to be HPV-related, 15,497 of which are attributable exclusively to HPV types 16 and 18 vs 32,562 which are expected to be related to these two types in women [19]. In addition it has also been estimated that there will be around 650,000 new cases each year of genital warts, more than 50% of which are expected in men. Similar data are confirmed by the most recent WHO (World Health Organization) statistical report on HPV in Europe [46]. A recent study conducted by Baio et al. [47] showed that the burden of HPV-related disease in Italy behaves similarly to that in Europe, with males playing an important role. The latest CDC data [18] on burden of disease in males display the same trend as Hartwig et al. [19] with almost half the number of cases of HPV-related cancer as women determined in Europe. Overall an average of 33,369 HPV-associated cancers (10,8 per 100.000 population), were diagnosed annually: 21,290 among women (13,2 per 100.000 population) and 12,080 among men (8,1 per 100.000 population). Cervical cancer was the most common, and oropharyngeal cancer ranked as the second most common. HPV-related head and neck cancer incidence in the USA is already higher in men, and should no action be taken, is expected to exceed that of cervical cancer by 2020 [18]. Anal cancer is a rare cancer; however, it has a very high incidence in men who have sex with men (MSM), where the incidence is estimated to be equivalent to that in women with cervical cancer, ranging from 32.8% to 93.5% [48-50], with an estimated risk of anal cancer 30 times [51,52] and genital warts around 10 times [53] higher than in heterosexual men. However not all cancers termed “HPV-associated” reflect actual infections and the numbers judged to be HPV-attributable are only estimates. The same MMWR editorial concludes: “Population-based screening for non-cervical HPV-associated cancers is not recommended” [18].

### Critical issues in HPV vaccination

Immunogenicity, safety, and clinical significance represent the critical issues on which to base the prospective of the eradicating HPV-related diseases through a global implementation of multivalent HPV vaccination.

Two vaccines are currently available to prevent HPV infection. The bivalent vaccine (HPV2) targets HPV types 16 and 18 [54], while the quadrivalent vaccine (HPV4) targets HPV types 6, 11, 16, and 18 [55]. Both vaccines have demonstrated high efficacy in the prevention of cervical precancerous lesions, long-term immunogenicity and efficacy, and to be safe and well tolerated in females up to 25 years of age. HPV4 vaccine has also demonstrated, together with the protection against cervical cancer, high efficacy against genital warts from HPV types 6 and 11, against vaginal and vulvar precancerous lesions, re-infection, persistent infection, and anal precancerous lesions (AIN 1,2,3 studied in an MSM population) [56-58]. HPV4 vaccine is also indicated for the use in females up to 45 years of age and in males up to 26 years of age by the European Medicines Agency (EMA) in Europe. Both vaccines are currently employed in girls in many national vaccination campaigns and have proven to be safe, well tolerated, and highly efficacious in preventing persistent infections and cervical diseases associated with specific HPV types among females [59]. Studies about long-term duration of efficacy are still ongoing for both vaccines, and are around 9 years without breakthroughs. Time is needed to clarify the issues of long-term duration of efficacy and immunogenicity and whether a vaccine boost will be required or not [60-62]. Through mathematical modeling Fraser et al. evaluated the long-term anti-HPV-16 responses following administration of a three-dose regimen of HPV-16 vaccine in women aged 16-23 years. Using a conventional power law model a median duration of detectable antibody (> 5.9 mMU/mL) of 32 years has been estimated; whereas the modified power law model predicted a long-term plateau of antibody duration with a nearly life-long persistence above the level of detection (> 5.9 mMU/mL) [63].

As mentioned before, the HPV burden of disease has been demonstrated to be quite high in men as well. Recent randomized studies have been conducted in order to assess efficacy, immunogenicity, and safety of the quadrivalent vaccine in men and included 4,065 young male subjects aged between 16 and 26 years of age, 602 of which self-declared to have sex with other men. Vaccine efficacy against external genital lesions (EGL) was found to be 90.4% and 89.4% against genital warts [56] (Table 1). The study by Giuliano and colleagues showed efficacy of 92.4% against genital warts in the heterosexual male population and 79% in the MSM population; for persistent infection the vaccine proved to have an efficacy of 50.4% for heterosexual males and 43.6% for MSM [56]. The same trial conducted to evaluate any grade of anal intraepithelial neoplasia (AIN) in MSM showed an overall efficacy on the per protocol population of 77.5% [57]. An exclusively post-hoc efficacy analysis for vaccine types of 92% was reported [58]. The studies [56-58] on male vaccine efficacy are summarized in Table 2. Efficacy of vaccine was bridged to males 10-15 years old with an immune-bridging study that demonstrated non-inferiority immune response compared to females aged 16 to 23 [64]. These studies are the basis for vaccine approval for males and male vaccination recommendations by EMA and FDA (Food and Drug Administration).

**Table 1:** **HPV4 vaccine efficacy study against external genital lesions and persistent HPV infection in men 16-26 years of age (n = 4065) **[56]

**Endpoint**	**HPV4 (n = 1397)**	**Placebo (n = 1408)**	**Efficacy (%)**	**95% CI**
	**N° cases**	**N° cases**		
All external genital lesions	3	31	90.4 (all HPV types)	62.2-97.9
Condyloma	3	28	89.4	65.5-97.9
Penile/perianal/perineal intraepithelial neoplasia	0	3	100	0-100
Persistent infection (all HPV types)	15	101	85.6	73.4-92.9
DNA detection	136	241	44.7	31.5-55.6

**Table 2:** **Summary of HPV4 vaccine efficacy studies in men **[56-58]

	**Giuliano**	**Palefsky**	**Goldstone**
Population	Per-protocol	Per-protocol	Per-protocol*
	(16-26 years)	(16-26 years)	(16-26 years)
External genital lesions	90.4%		
	(95% CI: 69.2-98.1)		
Anal intraepithelial neoplasia		77.5%	91.7%
		(95% CI: 39.6-93.3)	(95% CI: 44.6-99.8)
Condylomata acuminata	89.4%		
	(95% CI: 65.5-97.9)		

* Post-hoc analysis.

HPV vaccines have been demonstrated to be safe over the last 10 years. Most available data is on women, as they were the primary target of vaccination. Data on immunogenicity and safety are available however also for men and demonstrated that both HPV2 and HPV4 have immunogenicity and favorable safety profile similar to the studies conducted in women [65]. Both HPV vaccines are closely monitored worldwide and post-licensure studies have shown good safety profiles [66,67].

The clinical significance of HPV vaccination has been extensively studied in specific communities.

In the province of Victoria (Australia) an ecological study compared the incidence of high-grade pre-neoplastic and cancers (CIN 2+) lesions detected in women < 18 years of age examined before and after the start of an HPV4 vaccination program in young girls aged 12-to-13 years old [16]. A progressive decrease in the incidence of high-grade lesions by 0.38% has been observed in the girls younger than 18 years in a region where the vaccination coverage was between 71% and 79%. Another Australian ecological follow-up study [17] showed that after 5 years from the beginning of the vaccination program, genital incidence of new cases of genital wart dropped by 93%. Some herd immunity was also observed in males of the same age group, although incidence remained high in the MSM population [17]. Two other studies conducted in New Zealand [68] and California (USA) [69] – where the coverage of vaccination programs of the target population was rarely over 50% – showed around 60% and 30% reduction of genital warts, respectively. Evidence seems to support vaccine efficacy and reduction of disease with some herd immunity effect in heterosexual males but not in MSM. A rapid and marked reduction in the incidence of genital warts occurred among vaccinated women, and this reduction could mean some benefit being conferred to heterosexual men [70], but not to MSM. A rapid decline in presentation of genital warts was observed after implementation of a national program with HPV4 vaccine [70]. Given the success of Australia’s catch-up program, it will not be long before we know if the basic reproductive number for genital warts holds the prospect of elimination. However, if genital warts stabilize at a lower, but not very low, rate we will know that elimination will not be possible without vaccination of males [71].

### International policies and recommendations

Vaccination and vaccines are undoubtedly one of the most innovative procedures with the greatest impact on population health protection. Well-implemented vaccine policies achieve almost complete eradication of diseases once thought to be lethal.

In Europe, recommendations for HPV vaccination in females have been introduced in nearly all Western European countries with some of them also offering national or regional funding programs. It was first introduced in 2007 in Belgium, France, Germany, and Italy. In 2008 other countries also recommended vaccination, such as Greece, Luxembourg, the Netherlands, Romania, Spain, Switzerland, and the UK. Other European countries followed (Denmark, Norway, Portugal, San Marino, and Macedonia). The latest to start were Sweden and Ireland in 2010. As of today the vaccination advisory boards in 21 of the 29 countries of the EU have recommended and have in place active HPV vaccination programs [72]. In other countries (Czech Republic, Slovenia, Latvia, and Iceland), HPV vaccination has been recommended but has not been actually integrated in the national immunization programs. Some countries not only initiated their programs with the primary cohort of females but also have implemented different vaccination cohorts or catch-up programs. In Italy, the Basilicata region started in 2007/08 with a 4-cohort strategy covering females up to 25 years of age and assuring coverage of all females up to 21 years of age in 2012. This has resulted in average coverage rates in the primary cohort of around 80% and represents an excellent paradigm. Data collected by the VENICE2 Group in 2010 [72] registered a high heterogeneity in the strategies for implementation of the HPV vaccination in European countries. Recommendations for the vaccination starting age range from 10 to 18 and the catch-up rounds from age 12 to 24. So far the targets are girls/women in all European countries except Austria, which already targets but does not fund vaccination in males as well as females.

Today, in fact, the HPV4 vaccine is also indicated by the main health organizations (e.g. FDA, EMA) for males up to 26 years of age.

The US initially recommended routine vaccination of females between 11 and 12 years of age, with catch-up programs up to 26 years of age. Although the private American health system is not comparable to most public European health systems, the vaccine was offered for free both to the health-insured population and to the uninsured through Medicaid and the Vaccine for Children (VFC) program that offered the vaccine for free to girls up to 18 years of age. Even so, the US found several obstacles to reach high rates of vaccine coverage effectively in all regions, with an average coverage of only around 20% [73]. In 2011, the CDC Advisory Committee on Immunization Practices (ACIP) took into account new efficacy data on the vaccines, the low coverage rates, and the updated burden of disease knowledge and decided to recommend routine vaccination for both men and women, a gender-neutral vaccination offered free of charge, both to insured and uninsured people up to their 18^th^ birthday [74]. To support this decision, the American Academy of Pediatrics [75] recommended the vaccination of males as well as females. This new strategy is expected to further reduce the burden of the disease. In 2007 Canada and Australia which have national health systems decided to vaccinate women at the start of the program through school-based programs. Doing so, vaccine coverage reached higher coverage rates, ranging from 63.7% to 79.6% in Australia and from 50% to 85% in Canada [76-78]. The latest epidemiological studies in Australia show an almost complete disappearance of genital warts diagnosis: with a coverage of 83% of women < 21 years who received the first dose of the vaccine, the diagnosis of genital warts declined by 93% by the fifth year of the national quadrivalent HPV vaccination program [17]. In late 2011 and early 2012, both PBAC (Pharmaceutical Benefits Advisory Committee) and the NACI (National Advisory Committee on Immunization) [78] vaccine bodies in Australia and Canada have decided to recommend routine HPV vaccinations for males and females; the program started in Australia in January 2013.

Since the EMA approval in 2011 of male vaccination with the HPV4 vaccine, no European country (except Austria) has developed and implemented a program for routine vaccination in boys.

### Clinical effectiveness vs economic effectiveness

An overview of the results of many economic studies shows that vaccination against HPV produces immediate benefits from epidemiological points of view and in terms of avoided costs for country-specific National Health Systems (NHSs) [79-82]. Although estimates vary depending on the assumptions made, the cost-effectiveness of vaccination against HPV has been confirmed by a large body of modeling studies, which have been designed to evaluate different vaccination strategies [83,84]. In general, these studies compared a single cohort of women who underwent vaccination, plus optional catch-up cohorts, with women who underwent screening alone; the cohorts varied in age of immunization among studies. Studies evaluated the cost-effectiveness of vaccinating women of a particular age (e.g. 35 years) who had been participating in a specific screening strategy (e.g. biennial cytology) [85]. Two studies evaluated the implementation and economic consequences of a multi-cohort vaccination strategy [86,87]. At present, to be considered efficient from an economic point of view, health intervention should have an ICER (Incremental Cost Effectiveness Ratio) per QALY (Quality Adjusted Life Year) gained of less than £ 20,000 to £ 30,000 [88,89] (approximately € 30,000 to € 45,000). It should be noted that the threshold of £ 30.000 as defined by the National Institute for Health and Clinical Excellence (NICE) is not transferable to the specific policies on vaccines. The Canadian Agency for Drugs and Technologies in Health evaluated the cost-effectiveness of HPV vaccination in women and calculated a cost per QALY gained amounting to € 14,224 for the HPV4 vaccine and € 21,540 for the HPV2 vaccine [89]. Another study calculated QALY gained for HPV4 vaccines in Italy of € 9,569 [84]. In a recent study [87], results from a multi-cohort (12, 15, 16, and 25 years old) vaccination strategy of women confirmed the cost-effectiveness of HPV vaccination.

Several economic evaluations have assessed various HPV vaccine scenarios for men and women, on a cost-effectiveness basis in terms of QALY gained [79-81]. Even so, most cost-effectiveness studies of male vaccination tend to be centered on exclusively reducing cervical cancer in women and lack further important economic considerations – only lately have some studies started to take into account other vaccine benefits.

The published mathematical models using both men and women are based on assumptions that are not fully evidence-based. There are few studies that include males and those that are available also do not include all HPV-related diseases in both sexes and the coverage and compliance rates. Increasing coverage rates in females is a real challenge in most countries and is unlikely to be realized, unless a considerable budget effort is made by health authorities (public health campaigns, etc). The CDC recommendation (in November 2011) that young boys, as well as girls, should get immunized against HPV was based on the statement that male inclusion is cost-effective when coverage rates in females are low, such as is the case in the United States. In Australia, where the HPV vaccine coverage in female is high (> 80%), the decision to include routine vaccination of boys 12/13 years of age (recommended by the Pharmaceutical Benefits Advisory Committee (PBAC) [90]) was supported by showing that male inclusion could be cost-effective in Australia [91]. Following the Australian example, in January 2012 the National Advisory Committee on Immunization (NACI) in Canada recommended extension of the HPV4 vaccine to males between 9 and 36 years of age and routine vaccination of 12-year-old boys [78]. When more non-cervical diseases are included, cost per QALY gained decreased significantly. Moreover, not incorporating the reduction of number of non-cervical cancer cases in both sexes leads to a substantial underestimation of the cost per QALY gained of extending the vaccination to males. Elbasha et al. [92] presented all epidemiological outcomes and the number of cases avoided by vaccinating males in addition to females. The most oncogenic HPV type causing the greatest burden may be the most difficult to eliminate through vaccination of girls only; more substantial incremental benefits are expected by adding boys to vaccination programs when the HPV vaccine coverage is < 50% among young girls [93]. The cost/effectiveness of the impact on the overall population of including routine vaccine to MSM is still to be better defined. Boys and girls will mostly benefit from HPV vaccination if vaccinated routinely before becoming sexually active. In addition to protecting heterosexual males and their female partners, routine vaccination of boys at a young age is also the best way to reach MSM at an age when they could most benefit [74]. Furthermore, in the early stages of sexual life for a significant number of MSM, heterosexual activity is not uncommon thus contributing to the transmission of the virus [94]. Burden of disease in men seems to be quite significant, because genital warts alone have an important economic and psychological burden. Models taking into consideration several factors such as other HPV-related diseases seem to prove that male vaccination is expected to be cost effective.

### Introduction to male vaccination

One of the main goals of vaccination programs is to stop the transmission of an infective agent. In the case of HPV a single gender vaccination will not achieve such a goal. Moreover, why should vaccination programs target only one single disease such as cervical cancer, when vaccine benefits have also been proven to be high against other HPV-related diseases? Vaccinating boys is expected to facilitate the eradication of the cervical cancer, reduce the transmission of the virus, increase herd immunity, and contribute to the prevention of HPV-associated diseases in both genders. In fact more incremental benefits are expected by adding the boys in the vaccine program [93].

Neither the EMA nor the European Centre for Disease Prevention and Control (ECDC) guidelines [95] formally recommend vaccinating programs in boys or men. Among the scientific endorsements of an inclusion of males into vaccination programs for females, two different multidisciplinary panels of experts were convened between 2010 and 2012 in Italy. The Italian Society of Andrology, the Italian Society of Urologists, and the Italian Society of Andrology and Sexual Medicine created a panel of experts which developed a consensus statement arguing that the vaccine should be offered to males [96]. The second panel developed a commitment platform among scientists that is the basis of this paper. Italy is now also offering the vaccine on demand to males with a strategy of co-payment although informational and promotional campaigns have not been finalized yet.

Men play a key role in the paradigm of HPV infection: both as patients and as part of the mechanisms of transmission. As mentioned before, data show men are affected almost as frequently as women. Moreover no screening procedures for HPV prevention are currently ongoing in men, who do not receive routine medical testing by any medical specialist; in general the attitude in men towards prevention is low. They also do not benefit from government prevention strategies. It is well known that flat penile lesions play an important role in the transmission of high risk HPVs. In women the anal mucosa is a reservoir of HPV, which can be a source of re-infection for the cervix [97]. However, as observed in the mentioned study, there was not a significant association between the anal sex practice and the prevalence of anal cytological abnormalities. Including men in HPV vaccination programs increases its ranking on the list of urgent decisions to be taken by policy makers [98-101]. Information on HPV-related diseases is low or lacking in men; however, from studies focused on the acceptance by parents of vaccination of boys, it appears they would be willing to vaccinate their male children [102-104] and inclusion of men in the vaccine program will also increase coverage in women [105-107]. Discussion regarding the introduction of male vaccination is ongoing. Several key points could help in the extension of HPV vaccination programs to men such as: female-only vaccination will not protect all men; HPV-related head and neck cancer burden is carried mainly by men; it is the fastest way to achieve female protection by means of herd-immunity; vaccinating males is a more gender-equitable public health policy; men seem to accept vaccination as do parents of boys; vaccine seems to elicit the same if not a higher degree of immunogenicity in boys than in girls; genital warts and HPV-related cancers in men represent costly and emotionally burdensome and preventable conditions; lessons from the past with other vaccines show that single-gender-based vaccination policies are less effective.

*“…there is a good chance of drastically reducing cervical cancer by vaccination…. HPV-16 and -18 could probably be eliminated if we have a global program. You could theoretically achieve this by vaccinating only girls, but you would need very high coverage. I’m a strong advocate for vaccinating boys as well: we’ll reach the goal much faster by vaccinating both sexes. The disadvantage is that the cost is very high…”*[108]*.*

The HPV4 vaccine has proved to be effective and safe in men, but whether this is enough to recommend inclusion of males in NHS prevention strategies is still being debated. Several factors such as vaccine efficacy, herd immunity, vaccine coverage rates in females, burden of disease in men, and cost-benefit ratios need to be further evaluated when including men into the formula.

## Summary

### Achievements, pending questions, next steps

Vaccines are among the few medical interventions capable of achieving almost complete eradication of a disease. Today’s available epidemiological data show that HPV do not affect men and women differently and that men carry a considerable burden of the disease, enough to justify being included in national recommendations for immunization programs against HPV-associated lesions. Both the EMA and the FDA have approved HPV4 vaccine indication in males 9-26 years of age. Some national public health authority boards, such as in the USA, Canada, and Australia, already recommend men being included in their anti-HPV national routine immunization programs. So far, except for Austria, it is not yet recommended in Europe.

Taking advantage of the increasing opportunity to reduce HPV infection and transmission among sex partners, and of the increasing evidence on the effectiveness/efficacy of the HPV vaccines in preventing the development of HPV-related diseases, will decrease the burden of disease and increase the quality of life in the communities.

Including boys in vaccination programs can produce more incremental benefits globally to the currently unsolved and severe problem of HPV infection [93,109].

Those achievements pose questions for decision-makers as to their duty to overcome any barrier that impedes the achievement of health protection for both women and men against HPV infection. The issue of social equity in healthcare for both men and women is also one that must be addressed.

Future evaluation by the decision-makers in various countries of the results obtained by the next generation of intervention programs will focus on the critical issues that still exist: a) previous experience in gender-restricted vaccination programmes has demonstrated a substantially lower effectiveness than universal vaccination;b) limiting vaccination to women might increase the psychological burden on women by confirming a perceived inequality of the sexes; c) even if all women were immunized, the HPV chain of transmission would still be maintained through MSM; d) the cost-effectiveness of including boys in HPV vaccination programs should be re-assessed in view of the increased reduction, due to universal vaccination, of the economic burden of HPV related diseases in men and women [110].

Therefore steps must be taken by recommendation bodies and stakeholders to achieve the expected results of universal vaccination - the eradication of HPV infection. The goal to eradicate sexually transmitted carcinogenic viruses can be accomplished jointly by women and men within a few decades [111].

## Abbreviations

ACIP: Advisory committee on immunization practices; AGW: Ano genital warts; AIN: Anal intraepithelial neoplasia; CDC: Centers for disease control and prevention; CIN: Cervical intraepithelial neoplasia; ECDC: European centre for disease prevention and control; EGL: External genital lesions; EMA: European medicines agency; FDA: Food and drug administration; HPV: Human papilloma virus; HPV2: HPV bivalent vaccine; HPV4: HPV quadrivalent vaccine; IARC: International agency for research and cancer; MSM: Men who have sex with men; NACI: National advisory committee on immunization; NHSs: National health systems; PBAC: Pharmaceutical benefits advisory committee; QALY: Quality adjusted life year; VENICE: Vaccine european new integrated collaboration effort; VFC: Vaccine for children; WHO: World health organization.

## Competing interests

The authors declare that they have no competing interests.

## Authors’ contributions

The manuscript is a result of numerous discussions among the authors during three Workshops organized and moderated by the Fondazione Giovanni Lorenzini Medical Science Foundation (Milan, Italy and Houston, TX, USA). All authors have participated in writing the manuscript. All the authors have read and approved the final manuscript.

## Pre-publication history

The pre-publication history for this paper can be accessed here:

http://www.biomedcentral.com/1471-2458/13/642/prepub

## References

[B1] Baseman JG Koutsky LA The epidemiology of human papilloma virus infections J Clin Virol 2005 32 Suppl 1 S16 S24 15753008 10.1016/j.jcv.2004.12.008

[B2] Kahn JA Burk RD Papillomavirus vaccines in perspective Lancet 2007 369 2135 2137 10.1016/S0140-6736(07)60947-7 17602733

[B3] Bruni L Diaz M Castellsagué X Ferrer E Bosch FX de Sanjosé S Cervical human papillomavirus prevalence in 5 continents: meta-analysis of 1 million women with normal cytological findings J Infect Dis 2010 202 12 1789 1799 10.1086/657321 21067372

[B4] Syrjanen KJ Annual disease burden due to human papillomavirus 16 and 18 infections in Finland Scand J Infect Dis Suppl 2009 108 2 32 19939209 10.3109/00365540903331985

[B5] Zur Hausen H de Villiers EM Gissmann L Papillomavirus infections and human genital cancer Gynecol Oncol 1981 12 S124 S128 10.1016/0090-8258(81)90067-6 6273261

[B6] Zur Hausen H The search for infectious causes of human cancers where and why Virology 2009 392 1 1 10 10.1016/j.virol.2009.06.001 19720205

[B7] Tota J Chevarie-Davis M Richardson LA Devries M Franco EL Epidemiology and burden of HPV infection and related diseases: implications for prevention strategies Prev Med 2011 53 S12 S21 21962466 10.1016/j.ypmed.2011.08.017

[B8] Genden EM Sambur IM de Almeida JR Posner M Rinaldo A Rodrigo JP Strojan P Takes RP Ferlito A Human papillomavirus and oropharyngeal squamous cell carcinoma: what the clinician should know Eur Arch Otorhinolaryngol 2013 270 2 405 416 10.1007/s00405-012-2086-4 22752642

[B9] LaCour DE Trimble C Human papillomavirus in infants: transmission, prevalence, and persistence J Pediatr Adolesc Gynecol 2012 25 93 97 10.1016/j.jpag.2011.03.001 21600804 PMC3632362

[B10] Unger ER Fajman NN Maloney EM Onyekwuluje J Swan DC Howard L Beck-Sague CM Sawyer MK Girardet RG Sautter RL Hammerschlag MR Black CM Anogenital human papillomavirus in sexually abused and non abused children: a multicenter study Pediatrics 2011 128 3 e658 e665 21844060 10.1542/peds.2010-2247

[B11] Jones V Smith SJ Omar HA Nonsexual transmission of anogenital warts in children: a retrospective analysis ScientificWorldJournal 2007 7 1896 1899 18060328 10.1100/tsw.2007.276 PMC5901196

[B12] Honor G Ano-genital in children: sexual abuse or not? J Pediatr Health Care 2004 18 4 165 170 15224040 10.1016/j.pedhc.2003.01.001

[B13] Fairley CK Gay NJ Forbes A Abramson M Garland SM Hand-genital transmission of genital warts? An analysis of prevalence data Epidemiol Infect 1995 115 169 176 10.1017/S0950268800058234 7641831 PMC2271568

[B14] Sonnex C Strauss S Gray JJ Detection of human papillomavirus DNA on the fingers of patients with genital warts Sex Transm Inf 1999 75 317 319 10.1136/sti.75.5.317 PMC1758241 10616355

[B15] Rachel L Winer RL Hughes JP Feng Q Fu Xi L Cherne S O’Reilly S Kiviat NB Koutsky LA Detection of genital HPV types in fingertip samples from newly sexually active female university students Cancer Epidemiol Biomarkers Prev 2010 19 7 1682 1685 10.1158/1055-9965.EPI-10-0226 20570905 PMC2901391

[B16] Brotherton JM Fridman M May CL Chappell G Saville AM Gertig DM Early effect of the HPV vaccination programme on cervical abnormalities in Victoria, Australia: an ecological study Lancet 2011 377 2085 2092 10.1016/S0140-6736(11)60551-5 21684381

[B17] Ali H Donovan B Wand H Read TRH Regan DG Grulich AE Fairley CK Guy RJ Genital warts in young Australians five years into national human papillomavirus vaccination programme: national surveillance data BMJ 2013 346 f2032 Published 19 April 2013 10.1136/bmj.f2032 23599298

[B18] Morbidity and Mortality Weekly Reports MMWR 2012 61 No.15 258 261 http://www.cdc.gov/mmwr/pdf/wk/mm6115.pdf 22513527

[B19] Hartwig S Syrjanen S Dominiak-Felden G Brotons M Castellsaguè X Estimation of the epidemiological burden of human papillomavirus-related cancers and non-malignant diseases in men in Europe: a review BMC Cancer 2012 12 30 10.1186/1471-2407-12-30 22260541 PMC3293758

[B20] Gravitt PE The known unknowns of HPV natural history J Clin Invest 2011 121 12 4593 4599 10.1172/JCI57149 22133884 PMC3225991

[B21] Frazer IH Prevention of cervical cancer through papillomavirus vaccination Nature Rev Immunol 2004 4 46 55 10.1038/nri1260 14704767

[B22] Schiffman M Castle PE Jeronimo J Rodriguez AC Wacholder S Human papillomavirus and cervical cancer Lancet 2007 370 9590 890 907 10.1016/S0140-6736(07)61416-0 17826171

[B23] Frazer IH Measuring serum antibody to human papillomavirus following infection or vaccination Gynecol Oncol 2010 118 Issue 1, Supplement 1 S8 S11 20494221 10.1016/j.ygyno.2010.04.003

[B24] Franco E Villa LL Sobrinho JP Prado JM Rousseau MC Desy M Rohan TE Epidemiology and natural history of human papillomavirus infection in women from a high-risk area of cervical cancer J Infect Dis 1999 80 5 1415 1423 10515798 10.1086/315086

[B25] Woodman CB Collins S Winter H Bailey A Ellis J Prior P Yates M Rollason TP Young LS Natural history of cervical human papillomavirus infection in young women: a longitudinal cohort study Lancet 2001 357 9271 1831 1836 10.1016/S0140-6736(00)04956-4 11410191

[B26] Goldstein MA Goodman A del Carmen MG Wilbur DC Case records of the Massachusetts General Hospital. Case 10-2009. A 23-year-old woman with an abnormal Papanicolaou smear NEJM 2009 360 13 1337 1344 10.1056/NEJMcpc0810837 19321871

[B27] Giuliano AR Tortelero-Luna G Ferrer E Burchell AN de Sanjose S Kjaer SK Munoz N Schiffman M Bosch FX Epidemiology of human papilloma virus infection in men, cancers other than cervical and benign conditions Vaccine 2008 26 Suppl 10 K17 K28 18847554 10.1016/j.vaccine.2008.06.021 PMC4366004

[B28] Monk BJ Tewari KS The spectrum and clinical sequelae of human papillomavirus infection Nov Gynecol Oncol 2007 107 2 Suppl 1 S6 S13 10.1016/j.ygyno.2007.07.076 18499914

[B29] Badaracco G Rizzo C Mafera B Pichi B Giannarelli D Rahimi SS Vigili MG Venuti A Molecular analyses and prognostic relevance of HPV in head and neck tumours Oncol Rep 2007 17 931 939 17342339

[B30] Klussmann JP Weissenborn SJ Wieland U Dries V Kolligs J Jungehuelsing M Eckel HE Dienes HP Pfister H Fuchs PG Prevalence, distribution, and viral load of human papillomavirus 16 DNA in tonsillar carcinomas Cancer 2001 92 2875 2884 10.1002/1097-0142(20011201)92:11<2875::AID-CNCR10130>3.0.CO;2-7 11753961

[B31] De Stefani A Boffano P Averono G Ramella A Pia F Bongioannini G Prevalence and characteristics of HPV infection in oropharyngeal cancer J Craniofac Surg 2013 24 1 e40 e43 10.1097/SCS.0b013e31826cfffa 23348332

[B32] Herfs M Hubert P Moutschen M Delvenne P Mucosal junctions: open doors for HPV and HIV infections? Trends Microbiol 2011 19 114 120 10.1016/j.tim.2010.12.006 21216598

[B33] Giuliano AR Lazcano-Ponce E Villa LL Flores R Salmeron J Lee JH Papenfuss MR Abrahamsen M Jolles E Nielson CM Baggio ML Silva R Quiterio M The human papillomavirus infection in Men (HIM) study: HPV prevalence and type-distribution among men residing in Brazil, Mexico, and the US Cancer Epidemiol Biomarkers Prev 2008 17 8 2036 2043 10.1158/1055-9965.EPI-08-0151 18708396 PMC3471778

[B34] Vardas E Giuliano AR Goldstone S Palefsky JM Moreira ED Jr Penny ME Aranda C Jessen H Moi H Ferris DG Liaw KL Marshall JB Vuocolo S Barr E Haupt RM Garner EI Guris D External genital human papillomavirus prevalence and associated factors among heterosexual men on 5 continents J Infect Dis 2011 203 58 65 10.1093/infdis/jiq015 21148497 PMC3086430

[B35] Marks M Gravitt PE Gupta SB Liaw KL Kim E Tadesse A Phongnarisorn C Wootipoom V Yuenyao P Vipupinyo C Rugpao S Sriplienchan S Celentano DD The association of hormonal contraceptive use and HPV prevalence Int J Cancer 2011 128 12 2962 2970 10.1002/ijc.25628 20734390

[B36] Smith E Rubenstein LM Haugen TH Hamsikova E Turek LP Tobacco and alcohol use increase the risk of both HPV associated and HPV independent head and neck cancers Cancer Causes Control 2010 21 1369 1378 10.1007/s10552-010-9564-z 20401530

[B37] Winer RL Hughes JP Feng Q O’Reilly S Kiviat NB Holmes KK Koutsky LA Condom use and the risk of genital human papillomavirus infection in young women NEJM 2006 354 25 2645 2654 10.1056/NEJMoa053284 16790697

[B38] Wawer MJ Tobian AA Kigozi G Kong X Gravitt PE Serwadda D Nalugoda F Makumbi F Ssempiija V Sewankambo N Watya S Eaton KP Oliver AE Chen MZ Reynolds SJ Quinn TC Gray RH Effect of circumcision of HIV-negative men on transmission of human papillomavirus to HIV-negative women: a randomised trial in Rakai Uganda. Lancet 2011 377 9761 209 218 10.1016/S0140-6736(10)61967-8 PMC3119044 21216000

[B39] Tobian AA Serwadda D Quinn TC Kigozi G Gravitt PE Laeyendecker O Charvat B Ssempijja V Riedesel M Oliver AE Nowak RG Moulton LH Chen MZ Reynolds SJ Wawer MJ Gray RH Male circumcision for the prevention of HSV-2 and HPV infections and syphilis NEJM 2009 360 13 1298 1309 10.1056/NEJMoa0802556 19321868 PMC2676895

[B40] Smith JS Melendy A Rana RK Pimenta JM Age-specific prevalence of infection with human papillomavirus in females: a global review J Adolesc Health 2008 43 S5 S25 18809145 10.1016/j.jadohealth.2008.07.009

[B41] Giuliano AR Lee JH Fulp W Villa LL Lazcano E Papenfuss MR Abrahamsen M Salmeron J Anic GM Rollison DE Smith D Incidence and clearance of genital human papillomavirus infection in men (HIM): a cohort study Lancet 2011 377 932 940 10.1016/S0140-6736(10)62342-2 21367446 PMC3231998

[B42] Smith JS Gilbert PA Melendy A Rana RK Pimenta JM Age-specific prevalence of human papilloma virus infection in males: a global review J Adolesc Health 2011 48 540 552 10.1016/j.jadohealth.2011.03.010 21575812

[B43] Greer CE Wheeler CM Ladner MB Beutner K Coyne MY Liang H Langenberg A Yen TS Ralston R Human papillomavirus (HPV) type distribution and serological response to HPV type 6 virus-like particles in patients with genital warts J Clin Microbiol 1995 33 8 2058 2063 7559948 10.1128/jcm.33.8.2058-2063.1995 PMC228335

[B44] Blomberg M Friis S Munk C Bautz A Kjaer SK Genital warts and risk of cancer-a Danish study of nearly 50,000 patients with genital warts J Infect Dis 2012 205 10 1544 1553 10.1093/infdis/jis228 22427679

[B45] Grulich AE Jin F Conway EL Stein AN Hocking J Cancers attributable to human papillomavirus infection Sex Health 2010 7 244 252 10.1071/SH10020 20719211

[B46] WHO/ICO Information Centre, Human Papillomavirus and Related Cancers. Summary Report Update Summary Report Update 2010 EUROPE http://www.who.int/hpvcentre

[B47] Baio GL Capone A Marcellusi A Mennini FS Favato G Economic burden of human papillomavirus-related diseases in Italy PLoS One 2012 7 11 e49699 10.1371/journal.pone.0049699 23185412 PMC3504125

[B48] Vajdic CM van Leeuwen MT Jin F Prestage G Medley G Hillman RJ Stevens MP Botes LP Zablotska I Tabrizi SN Grulich AE Anal human papillomavirus genotype diversity and co-infection in a community-based sample of homosexual men Sex Transm Infect 2009 85 330 335 10.1136/sti.2008.034744 19342375

[B49] Chin-Hong PV Vittinghoff E Cranston RD Buchbinder S Cohen D Colfax G Da Costa M Darragh T Hess E Judson F Koblin B Madison M Palefsky JM Age-specific prevalence of anal human papillomavirus infection in HIV-negative sexually active men who have sex with men: the EXPLORE study J Infect Dis 2004 190 2070 2076 10.1086/425906 15551204

[B50] Van der Snoek EM Niesters HG Mulder PG van Doornum GJ Osterhaus AD van der Meijden WI Human papillomavirus infection in men who have sex with men participating in a Dutch gay-cohort study Sex Transm Dis 2003 30 639 644 10.1097/01.OLQ.0000079520.04451.59 12897686

[B51] Daling JR Weiss NS Hislop TG Maden C Coates RJ Sherman KJ Ashley RL Beagrie M Ryan JA Corey L Sexual practices, sexually transmitted diseases, and the incidence of anal cancer NEJM 1987 317 973 977 10.1056/NEJM198710153171601 2821396

[B52] Daling JR Weiss NS Klopfenstein LL Cochran LE Chow WH Daifuku R Correlates of homosexual behavior and the incidence of anal cancer JAMA 1982 247 1988 1990 10.1001/jama.1982.03320390050042 7062503

[B53] Pirotta M Stein AN Conway EL Harrison C Britt H Garland S Genital warts incidence and healthcare resource utilisation in Australia Sex Transm Infect 2010 86 181 186 10.1136/sti.2009.040188 19965802

[B54] Cervarix: Summary of Product Characteristics http://www.medicines.org.uk/emc/medicine/20204/SPC/Cervarix

[B55] Gardasil: Summary of Product Characteristics http://www.medicines.org.uk/emc/medicine/19016/SPC/gardasil/

[B56] Giuliano AR Palefsky JM Goldstone S Moreira ED Jr Penny ME Aranda C Vardas E Moi H Jessen H Hillman R Chang YH Ferris D Rouleau D Bryan J Marshall JB Vuocolo S Barr E Radley D Haupt RM Guris D Efficacy of quadrivalent HPV vaccine against HPV infection and disease in males NEJM 2011 364 5 401 411 10.1056/NEJMoa0909537 21288094 PMC3495065

[B57] Palefsky J Giuliano AR Goldstone S Moreira ED Jr Aranda C Jessen H Hillman R Ferris D Coutlee F Stoler MH Marshall JB Radley D Vuocolo S Haupt RM Guris D Garner EI HPV vaccine against anal HPV infection and anal intraepithelial neoplasia NEJM 2011 365 1576 1585 10.1056/NEJMoa1010971 22029979

[B58] Goldstone S A case assignment methodology for determining quadrivalent HPV vaccine efficacy against AIN in men having sex with men26th International Papillomavirus Conference 2010 Montreal, Canada http://www.eurogin.com/2010/EUROGIN2010_Abstracts.pdf

[B59] Lu B Lu B Kumar A Castellsagué X Giuliano AR Efficacy and safety of prophylactic vaccines against cervical HPV infection and disease among women: a systematic review & meta-analysis BMC Infect Dis 2011 11 13 10.1186/1471-2334-11-13 21226933 PMC3034689

[B60] Rowhani-Rahbar A Alvarez FB Bryan JT Hughes JP Hawes SE Weiss NS Koutsky LA Evidence of immune memory 8,5 years following administration of a prophylactic human papillomavirus type 16 vaccine J Clin Virol 2012 53 3 239 243 10.1016/j.jcv.2011.12.009 22209292 PMC3279625

[B61] Kjaer SK An evaluation of the Long-term effectiveness, immunogenicity and safety of Gardasil™ EUROGIN 2011 http://www.eurogin.com/2011/Eurogin-2011-Abstracts.pdf

[B62] Romanowski B de Borba PC Naud PS Roteli-Martins CM De Carvalho NS Teixeira JC Aoki F Ramjattan B Shier RM Somani R Barbier S Blatter MM Chambers C Ferris D Gall SA Guerra FA Harper DM Hedrick JA Henry DC Korn AP Kroll R Moscicki AB Rosenfeld WD Sullivan BJ Thoming CS Tyring SK Wheeler CM Dubin G Schuind A Zahaf T Greenacre M Sgriobhadair A GlaxoSmithKline Vaccine HPV-007 Study Group Sustained efficacy and immunogenicity of the human papillomavirus (HPV)- 16/18 AS04-adjuvanted vaccine: analysis of a randomised placebo-controlled trial up to 6.4 years Lancet 2009 374 9706 1975 1985 19962185 10.1016/S0140-6736(09)61567-1

[B63] Fraser C Tomassini JE Xi L Golm G Watson M Giuliano AR Barr E Ault KA Modeling the long-term antibody response of a human papillomavirus (HPV) virus-like particle (VLP) type 16 prophylactic vaccine Vaccine 2007 25 4324 4333 10.1016/j.vaccine.2007.02.069 17445955

[B64] Block SL Nolan T Sattler C Barr E Giacoletti KE Marchant CD Castellsagué X Rusche SA Lukac S Bryan JT Cavanaugh PF Jr Reisinger KS Protocol 016 Study Group Comparison of the immunogenicity and reactogenicity of a prophylactic quadrivalent human papillomavirus (types 6, 11, 16, and 18) L1 virus-like particle vaccine in male and female adolescents and young adult women Pediatrics 2006 118 5 2135 2145 10.1542/peds.2006-0461 17079588

[B65] Moreira ED Jr Palefsky JM Giuliano AR Goldstone S Aranda C Jessen H Hillman RJ Ferris D Coutlee F Vardas E Marshall JB Vuocolo S Haupt RM Guris D Garner EI Safety and reactogenicity of a quadrivalent human papillomavirus (types 6,11,16,18) L1 viral like particle vaccine in older adolescents and young adults Hum Vaccin 2011 7 7 768 775 10.4161/hv.7.7.15579 21712645 PMC3219080

[B66] Block SL Brown DR Chatterjee A Gold MA Sings HL Meibohm A Dana A Haupt RM Barr E Tamms GM Zhou H Reisinger KS Clinical trial and postlicensure safety profile of a prophylactic human papillomavirus (types 6, 11, 16, and 18) L1 virus-like particle vaccine Pediatr Infect Dis J 2010 29 95 101 10.1097/INF.0b013e3181b77906 19952863

[B67] Bonanni P Cohet C Kjaer SK Latham NB Lambert PH Reisinger K Haupt RM A summary of the post-licensure surveillance initiatives for GARDASIL/SILGARD® Vaccine 2010 28 4719 4730 10.1016/j.vaccine.2010.04.070 20451636

[B68] Oliphant J Perkins N Impact of the human papillomavirus vaccine on genital wart diagnoses at Auckland Sexual Health Services NZMJ 2011 124 1339 51 58 21952330

[B69] Bauer HM Wright G Chow J Evidence of human papillomavirus vaccine effectiveness in reducing genital warts: an analysis of California public family planning administrative claims data, 2007-2010 Am J Public Health 2012 102 5 833 835 10.2105/AJPH.2011.300465 22420808 PMC3483904

[B70] Fairley CK Hocking JS Gurrin LC Chen MY Donovan B Bradshaw CS Rapid decline in presentations of genital warts after the implementation of a national quadrivalent human papillomavirus vaccination programme for young women Sex Transm Infect 2009 85 7 499 502 10.1136/sti.2009.037788 19837728

[B71] Fairley CK Donovan B **What can surveillance of genital warts tell us**? Sex Health 2010 7 3 325 327 10.1071/SH09145 20719222

[B72] Dorleans F Giambi C Dematte L Cotter S Stefanoff P Mereckiene J O’Flanagan D Lopalco PL D’Ancona F Lévy-Bruhl D on behalf of the VENICE 2 project gatekeepers group The current state of introduction of human papillomavirus vaccination into national immunisation schedules in Europe: first results of the VENICE2 2010 survey Euro Surveill 2010 15 47 Available online: http://www.eurosurveillance.org/ViewArticle.aspx?ArticleId=19730 10.2807/ese.15.47.19730-en 21144444

[B73] Morbidity and mortality weekly report (MMRW) adult vaccination coverage-United states 2010 2012 61 04 66 72 http://www.cdc.gov/mmwr/preview/mmwrhtml/mm6104a2.htm 22298302

[B74] Advisory Committee on Immunization Practices (ACIP) Recommendations on the use of quadrivalent human papillomavirus vaccine in males MMRW 2011 60 50 1707 1708 22189893

[B75] Brady MT Byington CL Davies HD Edwards KM Glode MP Jackson MA Keyserling HL Maldonado YA Murray DL Orenstein WA Schutze GE Willoughby RE Zaoutis TE HPV vaccine recommendations. American academy of paediatrics Pediatrics 2012 129 3 602 605 22371460 10.1542/peds.2011-3865

[B76] Australian Government. Department of Health and Ageing. Immunise Australia Program. Human papillomavirus (HPV). Australian Government [Accessed 31 July 2011]. [http://www.immunise.health.gov.au/internet/immunise/publishing.nsf/Content/immunise-hpv]

[B77] Gertig DM Brotherton JM Saville M Measuring human papillomavirus (HPV) vaccination coverage and the role of the national HPV vaccination program register Australia. Sex Health 2011 8 2 171 178 10.1071/SH10001 21592430

[B78] An Advisory Committee Statement (ACS) National Advisory Committee on Immunization (NACI). Update on Human Papillomavirus (HPV) Vaccines Canada Comunicable Disease Report (CCDR) 2012 37 1 62 10.14745/ccdr.v38i00a01 PMC6802461 31701955

[B79] Jit M Choi YH Edmunds WJ Economic evaluation of human papillomavirus vaccination in the United Kingdom BMJ 2008 337 a769 10.1136/bmj.a769 18640957 PMC2500202

[B80] Kim JJ Goldie SJ Cost effectiveness analysis of including boys in a human papillomavirus vaccination programme in the United States BMJ 2009 339 b3884 10.1136/bmj.b3884 19815582 PMC2759438

[B81] Taira AV Neukermans CP Sanders GD Evaluating human papillomavirus vaccination programs Emerg Infect Dis 2004 10 1915 1923 10.3201/eid1011.040222 15550200 PMC3328990

[B82] Chesson HW Ekwueme DU Saraiya M Markowitz LE Cost-effectiveness of human papillomavirus vaccination in the United States Emerg Infect Dis 2008 14 2 244 251 10.3201/eid1402.070499 18258117 PMC2600200

[B83] Kim JJ Goldie SJ Health and economic implications of HPV vaccination in the United States NEJM 2008 359 821 832 10.1056/NEJMsa0707052 18716299 PMC3080183

[B84] Mennini FS Giorgi Rossi P Palazzo F Largeron N Health and economic impact associated with a quadrivalent HPV vaccine in Italy Gynecol Oncol 2009 112 370 376 10.1016/j.ygyno.2008.09.031 19041125

[B85] Elbasha EH Dasbach EJ Insinga RP Model for assessing human papillomavirus vaccination strategies Emerg Infect Dis 2007 13 28 41 10.3201/eid1301.060438 17370513 PMC2725801

[B86] Favato G Pieri V Mills R Cost-effective analysis of anti-HPV vaccination programme in Italy: a multi-cohort Markov model 2007 Available at SSRN: http://ssrn.com/abstract=961847 or http://dx.doi.org/10.2139/ssrn.961847

[B87] Favato G Baio G Capone A Marcellusi A Costa S Garganese G Picardo M Drummond M Jonsson B Scambia G Zweifel P Mennini FS Novel health economic evaluation of a vaccination strategy to prevent HPV-related diseases Med Care 2012 50 12 1076 1085 10.1097/MLR.0b013e318269e06d 22922435

[B88] Rawlins MD Culyer AJ National institute for clinical excellence and its value judgments BMJ 2004 329 7459 224 227 10.1136/bmj.329.7459.224 15271836 PMC487742

[B89] Mennini FS Costa S Favato G Picardo M Anti-HPV vaccination: a review of recent economic data for Italy Vaccine 2009 27 A54 A61 19480963 10.1016/j.vaccine.2009.02.052

[B90] Nolan TM The Australian model of immunization advice and vaccine funding Vaccine 2010 28 A76 A83 20413003 10.1016/j.vaccine.2010.02.038

[B91] Kulasingam S Connelly L Conway E Hocking JS Myers E Regan DG Roder D Ross J Wain G A cost-effectiveness analysis of adding a human papillomavirus vaccine to the Australian national cervical cancer screening program Sex Health 2007 4 165 175 10.1071/SH07043 17931529

[B92] Elbasha EH Dasbach EJ Impact of vaccinating boys and men against HPV in the United States Vaccine 2010 28 6858 6867 10.1016/j.vaccine.2010.08.030 20713101

[B93] Brisson M van de Velde N Franco EL Drolet M Boily MC Incremental impact of adding boys to current human papillomavirus vaccination programs: role of herd immunity J Infect Dis 2011 204 3 372 376 10.1093/infdis/jir285 21742835

[B94] Reece M Herbenick D Schick V Sanders SA Dodge B Fortenberry JD Sexual behaviors, relationships, and perceived health among adult men in the United States: results from a national probability sample J Sex Med 2010 7 Suppl 5 291 304 21029386 10.1111/j.1743-6109.2010.02009.x

[B95] ECDC guidance. Introduction of HPV vaccines in European Union countries-an updatehttp://www.ecdc.europa.eu

[B96] Lenzi A Mirone V Gentile V Bartoletti R Ficarra V Foresta C Mariani L Mazzoli S Parisi SG Perino A Picardo M Zotti CM Rome consensus conference - statement; human papilloma virus diseases in males BMC Publ Health 2013 13 117 10.1186/1471-2458-13-117 PMC3642007 23391351

[B97] Calore EE Giaccio CM Nadal SR Prevalence of anal cytological abnormalities in women with positive cervical cytology Diagn Cytopathol 2011 39 5 323 327 10.1002/dc.21386 21488174

[B98] Weiss TW Zimet GD Rosenthal SL Brenneman SK Klein JD Human papillomavirus vaccination of males: attitudes and perceptions of physicians who vaccinate females J Adolesc Health 2010 47 1 3 11 10.1016/j.jadohealth.2010.03.003 20547286

[B99] Palefsky JM Human papillomavirus-related disease in men: not just a women’s issue J Adolesc Health 2010 46 4 Suppl S12 S19 20307839 10.1016/j.jadohealth.2010.01.010 PMC2871537

[B100] Goldstone SE Some straight talk about anal human papillomavirus infection J Infect Dis 2010 201 10 1450 1452 10.1086/652188 20367456

[B101] Oon SF Winter DC Perianal condylomas, anal squamous intraepithelial neoplasms and screening: a review of the literature J Med Screen 2010 17 1 44 49 10.1258/jms.2009.009058 20356945

[B102] Brewer NT Ng TW McRee AL Reiter PL Men’s beliefs about HPV-related disease J Behav Med 2010 33 274 281 10.1007/s10865-010-9251-2 20162346 PMC4018629

[B103] Hernandez BY Wilkens LR Thompson PJ Shvetsov YB Goodman MT Ning L Kaopua L Acceptability of prophylactic human papillomavirus vaccination among adult men Hum Vaccine 2010 6 467 475 10.4161/hv.6.6.11279 PMC3010761 20671442

[B104] Reiter PL Brewer NT McRee AL Gilbert P Smith JS Acceptability of HPV vaccine among a national sample of gay and bisexual men Sex Transm Dis 2010 37 197 203 10.1097/OLQ.0b013e3181bf542c 20118831 PMC4018212

[B105] Chesson HW Ekwueme DU Saraiya M Dunne EF Markowitz LE The cost-effectiveness of male HPV vaccination in the United States Vaccine 2011 29 46 8443 8450 10.1016/j.vaccine.2011.07.096 21816193

[B106] Marty R Roze S Bresse X Largeron N Smith-Palmer J Estimating the clinical benefits of vaccinating boys and girls against HPV-related diseases in Europe BMC Cancer 2013 13 10 1 12 23298365 10.1186/1471-2407-13-10 PMC3561184

[B107] Baandrup L Blomberg M Dehlendorff C Sand C Andersen KK Kjaer SK Significant decrease in the incidence of genital warts in young Danish women after implementation of a national human papillomavirus vaccination program Sex Transm Dis 2013 40 2 130 135 23324976 10.1097/OLQ.0b013e31827bd66b

[B108] Grayson M From an interview to harald Zur Hausen Nature 2012 488 7413 16 10.1038/488S16a 22932436

[B109] Low MI Attiga YS Garg G Schelgal R Gllicano GI Can male vaccination reduce burden of human papillomavirus-related disease in the United States? Viral Immunol 2012 25 3 174 183 22691099 10.1089/vim.2011.0083

[B110] European Centre for Disease Prevention and Control Introduction of HPV vaccines in EU countries – an update 2012 Stockholm: ECDC 978-92-9193-377–8 10.2900/60814

[B111] Michels KB Zur Hausen H HPV vaccine for all Lancet 2009 374 9686 268 270 10.1016/S0140-6736(09)61247-2 19586657

